# Potent antiviral activity of simnotrelvir against key epidemic SARS-CoV-2 variants with a high resistance barrier

**DOI:** 10.1128/aac.01556-24

**Published:** 2025-03-10

**Authors:** Liwei Zhao, Chuang Li, Mengyu Wang, Minyun Zhou, Lei Jiang, Wanying Zhang, Jie Yu, Wei Wang, Kangping Zhou, Kai Pan, Hoi-Yan Lam, Ivan Fan-Ngai Hung, Kwok-Hung Chan, Lian Liu, Feng Wang, Xiaofeng Zhao, Yuxin Chen

**Affiliations:** 1 Department of Laboratory Medicine, Nanjing Drum Tower Hospital Clinical College of Nanjing University of Chinese Medicine 66506 , Nanjing, Jiangsu, China; 2 Jiangsu Simcere Pharmaceutical Company Limited 71168 , Nanjing, Jiangsu, China; 3 State Key Laboratory of Neurology and Oncology Drug Development, Nanjing, China; 4 Simcere Zaiming Pharmaceutical Company Limited, Shanghai, China; 5 Department of Chemical Engineering, Tsinghua University 351829 , Beijing, China; 6 Department of Laboratory Medicine, Nanjing Drum Tower Hospital Clinical College of Nanjing Medical University 12461 , Nanjing, Jiangsu, China; 7 Department of Laboratory Medicine, Nanjing Drum Tower Hospital, Nanjing University Medical School, Nanjing, Jiangsu, China; 8 Hubei Provincial Center for Disease Control and Prevention 498598 , Wuhan, Hubei, China; 9 Department of Microbiology, State Key Laboratory of Emerging Infectious Diseases, Carol Yu Centre for Infection,The University of Hong Kong, Hong Kong SAR, China; 10 Department of Medicine, Queen Mary Hospital, The University of Hong Kong, Hong Kong SAR, China; 11 Centre for Virology, Vaccinology and Therapeutics, Hong Kong Science and Technology Park, Hong Kong SAR, China; IrsiCaixa Institut de Recerca de la Sida, Barcelona, Spain

**Keywords:** simnotrelvir, 3C-like protease, SARS-CoV-2, antiviral efficacy, antiviral resistance

## Abstract

Simnotrelvir is an oral small-molecule antiviral agent targeting the 3C-like protease (3CL^pro^) of SARS-CoV-2, proven effective against the Delta variant with favorable pharmacokinetics and safety in preclinical study. In this study, we further evaluated the antiviral efficacy of simnotrelvir against a range of emerging Omicron variants, including BA.1, BA.4, BA.5, CH.1.1, XBB.1.5, XBB.1.16, EG.5, and JN.1. *In vitro* assays with Vero E6 cells confirmed that simnotrelvir exhibited robust antiviral activity across these variants, comparable to the Food and Drug Administration (FDA)-approved drug nirmatrelvir. Additionally, simnotrelvir demonstrated effective inhibition against several nirmatrelvir-resistant SARS-CoV-2 3CL^pro^ mutants, including A260V, Y54A, (T21I + S144A), F140A, H172Y, and E166V. Importantly, simnotrelvir showed better potency against the E166V mutation compared to nirmatrelvir. Resistance selection studies revealed that BA.5 developed reduced sensitivity after 5 and 10 passages, increasing the IC_50_ values by 3.2 and 4.5-fold, respectively, while HCoV-OC43 showed an 8.3-fold increase after 12 passages. Despite this, simnotrelvir’s overall efficacy remains strong. Furthermore, clinical trials demonstrated that combining simnotrelvir with ritonavir significantly shortened symptom resolution in COVID-19 patients. Genomic analysis of treated patients found random nucleotide substitutions but no significant mutations linked to 3CL^pro^ resistance. In conclusion, simnotrelvir shows strong antiviral activity against SARS-CoV-2 variants and maintains a high barrier to resistance, reinforcing its potential as an effective therapeutic option for current and future SARS-CoV-2 variants.

## INTRODUCTION

The severe acute respiratory syndrome coronavirus 2 (SARS-CoV-2) emerged at the end of 2019, causing severe acute respiratory coronavirus disease 2019 (COVID-19) and triggering a global pandemic (1, 2). Large-scale vaccination is considered crucial for controlling the COVID-19 pandemic. However, the emergence of new SARS-CoV-2 variants, such as KP.2 and KP.3, is rapidly increasing infection rates globally, becoming dominant strains in circulation (3). According to World Health Organization (WHO) surveillance data, as of 12 August 2024, the global infection rate has reached 21% (4). It is possibly due to the remarkable mutations of the Omicron variant and the waned immunity elicited by either infection or vaccination (3, 4). COVID-19 therapeutics, especially monoclonal antibodies, had lost their efficacy against newly emerging SARS-CoV-2 variants, highlighting the need for more effective antiviral drugs to treat COVID-19.

The coronavirus 3C-like protease (3CL^pro^), also known as main protease (M^pro^), highly conserved across a range of pathogenic coronaviruses, is responsible for the cleavage of two polyproteins (pp1a and pp1b) during viral replication (5–7), making 3CL^pro^ an attractive target for antiviral drug development. Oral small-molecule drugs offer the convenience of easy administration and are less sensitive to storage conditions, making them more accessible for patients. Additionally, they exhibit minimal immunogenicity, reducing the possibility of allergic reactions (8), which makes the production of oral small-molecule drugs targeting 3CL^pro^ a promising way to fight COVID-19. Currently, several 3CL^pro^ inhibitors have been approved for COVID-19 treatment, such as nirmatrelvir (FDA approved) (9), simnotrelvir (National Medical Products Administration [NMPA] approved) (10), ensitrelvir (Pharmaceuticals and Medical Devices Agency [PMDA] approved) (11) and leritrelvir (FDA approved) (12). Simnotrelvir is an oral small-molecule antiviral agent that also targets the SARS-CoV-2 3CL^pro^ for the treatment of mild-to-moderate COVID-19 in adult patients. It was approved for use under an emergency conditional authorization in Jan 2023 and received full approval in July 2024 (13). Preclinical studies have confirmed that simnotrelvir can inhibit the replication of SARS-CoV-2 variants at the cellular level, demonstrating favorable pharmacokinetics and safety profiles (14). Additionally, a phase 2–3 double-blind, randomized, placebo-controlled clinical trial has confirmed the efficacy and safety of simnotrelvir plus ritonavir in treating adult patients with COVID-19 (10).

The main feature of the SARS-CoV-2 variants is mutations in the spike protein, which can reduce the effectiveness of therapeutic monoclonal antibodies and vaccines (15–17). However, since simnotrelvir targets the highly conserved 3CL^pro^, it is predicted that simnotrelvir will remain active against SARS-CoV-2 variants with new spike protein mutations. Our previous studies have demonstrated that simnotrelvir is effective not only against the SARS-CoV-2 WIV04 and Delta but also against early Omicron variants, including B.1.1.529 (14). Therefore, we further investigated the inhibitory activity of simnotrelvir against newly emerging SARS-CoV-2 variants of concern (VOC), including BA.1, BA.4, BA.5, CH.1.1, XBB.1.5, XBB.1.16, EG.5, and JN.1.

With the increasing clinical use of 3CL^pro^ inhibitors, the emergence of drug resistance has become a growing concern (18–22). In order to assess whether simnotrelvir has a high resistance barrier to the nirmatrelvir-resistant SARS-CoV-2 3CL^pro^ mutants, we first evaluated the enzymatic inhibition effect of simnotrelvir against various 3CL^pro^ mutants *in vitro*. Furthermore, we evaluated the changes in the susceptibility of SARS-CoV-2 BA.5 or HCoV-OC43 to simnotrelvir after serial passages of the virus in the presence of simnotrelvir. Ultimately, the genotypic changes of SARS-CoV-2 were analyzed from simnotrelvir-treated COVID-19 patients to identify the emergence of clinical resistance mutations. Taken together, our data highlight that simnotrelvir exhibited potent antiviral activity against SARS-CoV-2 variants and a higher barrier for drug resistance.

## RESULTS

### Simnotrelvir showed potent antiviral activity against new emerging SARS-CoV-2 Omicron variants

**Fig 1:**
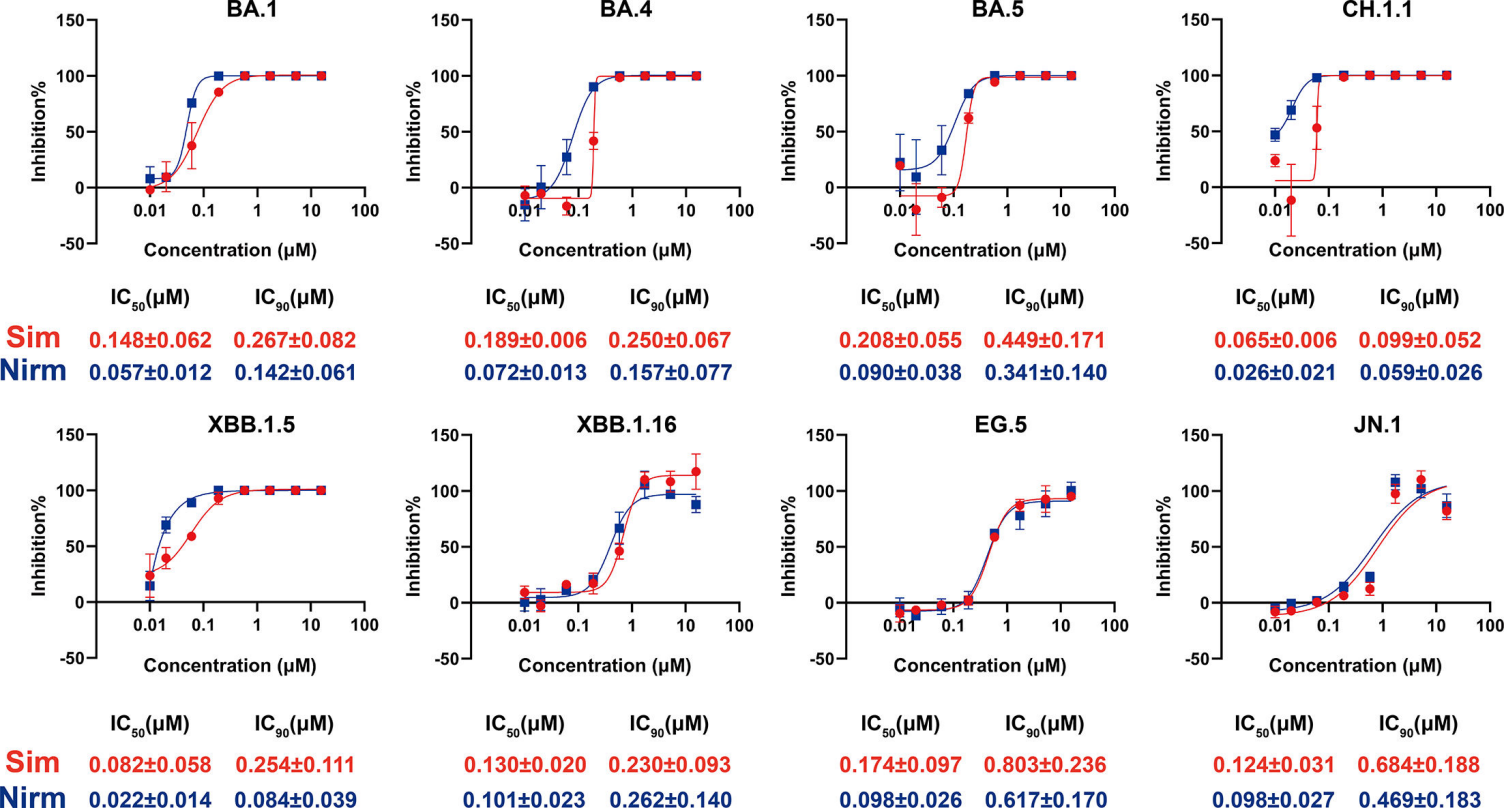
*In vitro* cellular antiviral activity of simnotrelvir Vero E6 cells treated with simnotrelvir or nirmatrelvir and infected with various SARS-CoV-2 Omicron variants. *In vitro* 50% inhibitory concentration (IC_50_) and 90% inhibitory concentration (IC_90_) were determined. The final concentration of 0.5 µM P-gp inhibitor (CP-100356) was added during the experimental process. Concentration response inhibition curves for simnotrelvir and nirmatrelvir against multiple SARS-CoV-2 Omicron variants. The red curve indicates simnotrelvir, while the blue curve represents nirmatrelvir. Representative curves from a single experiment from three biologically independent experiments are shown. Error bars denote mean  ±  SD of three technical replicates.

We have shown that simnotrelvir exhibited potent inhibitory activities against SARS-CoV-2, including SARS-CoV-2 WIV04 (IC_50_: 0.026 µM), Delta (IC_50_: 0.034 µM), and the Omicron variant B.1.1.529 (IC_50_: 0.043 µM) (14). To confirm whether simnotrelvir remained effective against the emerging variants, we evaluated its inhibitory activities against eight new emerging Omicron variants by measuring the 50% inhibitory concentration (IC_50_) and 90% inhibitory concentration (IC_90_) values of simnotrelvir in Vero E6 cells. As shown in Fig. 1, simnotrelvir maintained strong antiviral activity against all evaluated SARS-CoV-2 Omicron variants, including BA.1 (IC_50_ = 0.148 ± 0.062 µM, IC_90_ = 0.267 ± 0.082 µM), BA.4 (IC_50_ = 0.189 ± 0.006 µM, IC_90_ = 0.250 ± 0.067 µM), BA.5 (IC_50_ = 0.208 ± 0.055 µM, IC_90_ = 0.449 ± 0.171 µM), CH.1.1 (IC_50_ = 0.065 ± 0.006 µM, IC_90_ = 0.099 ± 0.052 µM), XBB.1.5 (IC_50_ = 0.082 ± 0.058 µM, IC_90_ = 0.254 ± 0.111 µM), XBB.1.16 (IC_50_ = 0.130 ± 0.020 µM, IC_90_ = 0.230 ± 0.093 µM), EG.5 (IC_50_ = 0.174 ± 0.097 µM, IC_90_ = 0.803 ± 0.236 µM), and JN.1 (IC_50_ = 0.124 ± 0.031 µM, IC_90_ = 0.684 ± 0.188 µM) (Fig. 1). Thus, simnotrelvir exhibited potent antiviral efficacy against eight new emerging SARS-CoV-2 Omicron variants, comparable to that of nirmatrelvir (the first approved 3CL^pro^ inhibitor for SARS-CoV-2 treatment as the benchmark compound in our assays).

### Simnotrelvir exhibited inhibitory effects against nirmatrelvir-resistant SARS-CoV-2 3CL^pro^ resistant mutants

**Fig 2:**
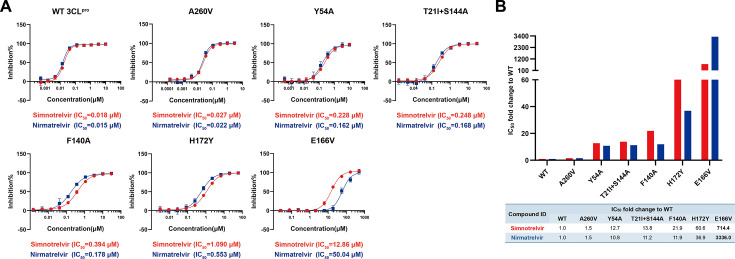
Inhibitory activity of simnotrelvir against nirmatrelvir-resistant 3CL^pro^ mutants. The inhibitory activity of simnotrelvir against wild-type 3CL^pro^ and nirmatrelvir-resistant 3CL^pro^ mutants was measured using a FRET-based assay. (**A**) Concentration response inhibition curves for simnotrelvir against WT 3CL^pro^ and six 3CL^pro^ mutants, and the IC_50_ values for them are displayed beneath the corresponding graph. Simnotrelvir is represented by red curves and text, while nirmatrelvir is indicated by blue curves and text. Error bars denote mean ± SD of three technical replicates. (**B**) The fold change in IC_50_ values of simnotrelvir and nirmatrelvir against six 3CL^pro^ mutants, compared to WT 3CL^pro^. Red bars represent simnotrelvir, and blue bars represent nirmatrelvir. The fold changes in IC_50_ values are detailed in the table below the figure.

Several SARS-CoV-2 3CL^pro^ mutants, including A260V (23), Y54A (23), (T21I + S144A) (24, 25), F140A (23), H172Y (26), and E166V (25), have recently been shown to be associated with varying degrees of resistance to nirmatrelvir *in vitro* or in clinical studies. To determine whether these mutants might also confer resistance to simnotrelvir, the enzymatic inhibition of simnotrelvir against these 3CL^pro^ mutants was tested. Simnotrelvir showed efficient inhibition against the six 3CL^pro^ mutants, namely, A260V (IC_50_: 0.027 µM), Y54A (IC_50_: 0.228 µM), (T21I + S144A) double mutations (IC_50_: 0.248 µM), F140A (IC_50_: 0.394 µM), H172Y (IC_50_: 1.090 µM), and E166V (IC_50_: 12.86 µM) (Fig. 2A). Based on our previous studies, the IC_50_ value of simnotrelvir against wild-type 3CL^pro^ was 0.018  µM, while the IC_50_ value of nirmatrelvir against wild-type 3CL^pro^ was 0.015 µM. Other than that similar to nirmatrelvir, six mutants displayed varying degrees of resistance to simnotrelvir, with 1.5-fold for A260V, 12.7-fold for Y54A, 13.8-fold for (T21I + S144A) double mutant, 21.9-fold for F140A, 60.6-fold for H172Y, and 714.4-fold for E166V. Notably, simnotrelvir exhibited approximately 4.0-fold greater sensitivity against E166V compared to nirmatrelvir**,** while showing comparable sensitivity levels for the remaining five 3CL^pro^ mutants (Fig. 2B). Therefore, our data suggest that simnotrelvir may maintain a relatively higher efficacy than nirmatrelvir against SARS-CoV-2 with E166V mutation.

### *In vitro* susceptibility of Omicron BA.5 variant and HCoV-OC43 to simnotrelvir following serial passages

**Fig 3:**
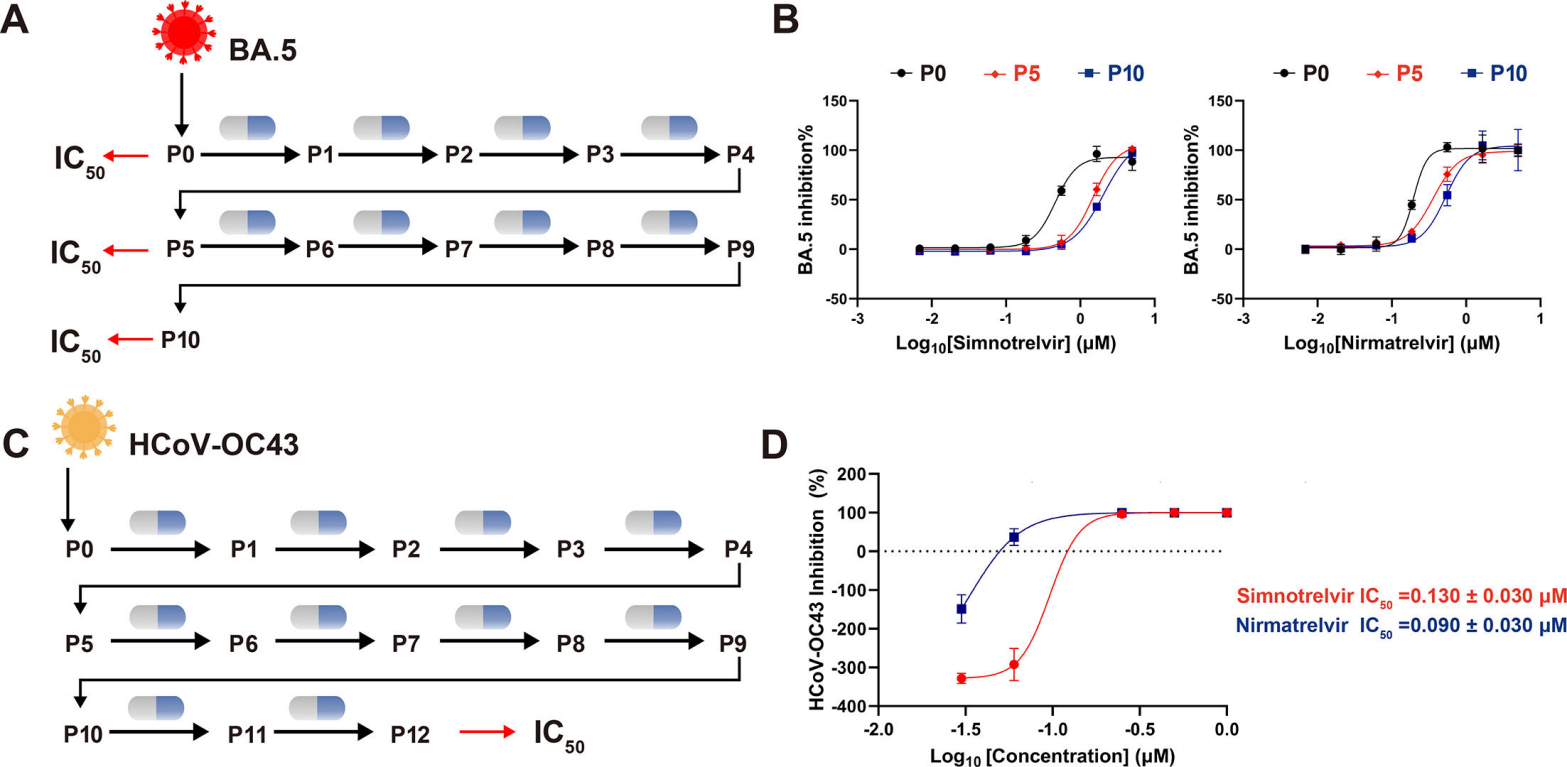
The sensitivity of passaged SARS-CoV-2 Omicron BA.5 and HCoV-OC43 to simnotrelvir (**A, C**) Schematic diagram of *in vitro* selection for the BA.5 variant or HCoV-OC43 resistance assay: SARS-CoV-2 Omicron BA.5 or HCoV-OC43 was co-cultured in the presence of simnotrelvir and passaged to fresh cells every 3–4  days. (**B**) Inhibition curves of P0, P5, and P10 SARS-CoV-2 Omicron BA.5 variants treated with simnotrelvir. Vero E6 cells were infected with BA.5 and passaged for 10 passages. After 0 passages (**P0**), five passages (**P5**), and 10 passages (**P10**), IC_50_ were detected. (**D**) Validation of HCoV-OC43 to simnotrelvir resistance in 12 passages. RD cells were infected with HCoV-OC43 and passaged to fresh cells every 3  days for 12 passages. Red represents simnotrelvir, and blue represents nirmatrelvir. Representative curves from a single experiment from three biologically independent experiments are shown. Error bars denote mean ± SD of three technical replicates.

To assess whether simnotrelvir treatment leads to any decreased susceptibility of SARS-CoV-2, the *in vitro* susceptibility assessments of serial-passaged Omicron BA.5 to simnotrelvir were conducted for BA.5 cultures from passages 5 and 10 using the Vero E6 cell inhibitory assay (Fig. 3A). The IC_50_ value of simnotrelvir against the 5-passaged BA.5 variant has a 3.2-fold increment (from 0.460 μM to 1.491 µM), and IC_50_ of simnotrelvir against the 10-passaged BA.5 variant was increased by 4.0-fold (from 0.460 μM to 1.820 µM), compared to non-passaged BA.5. Similarly, nirmatrelvir exhibited a 1.8-fold increase in IC_50_ values (from 0.196 μM to 0.357 µM) after five passages and a 2.7-fold increment (from 0.196 μM to 0.532 µM) after 10 passages (Fig. 3B).

Due to the essential role of 3CL^pro^ in viral replication and its high conservation across various β-coronavirus strains (7, 27, 28), we subsequently extended our analysis to alternative β-coronavirus, HCoV-OC43 (Fig. 3C). For 12-passaged HCoV-OC43, the IC_50_ of simnotrelvir increased by 8.3-fold (from 0.018 ± 0.006 µM to 0.130 ± 0.030 µM) (Fig. 3D)**,** whereas nirmatrelvir exhibited a 7.2-fold increase in IC_50_ values (from 0.013 ± 0.002 µM to 0.090 ± 0.030 µM). Despite the slight decrease in the potency, simnotrelvir still maintains a relatively high inhibitory effect against the passaged BA.5 and HCoV-OC43 virus *in vitro*, indicating potentially maintained efficacy *in vivo*.

### Simnotrelvir did not confer resistance to clinical resistant SARS-CoV-2 variants

**Fig 4:**
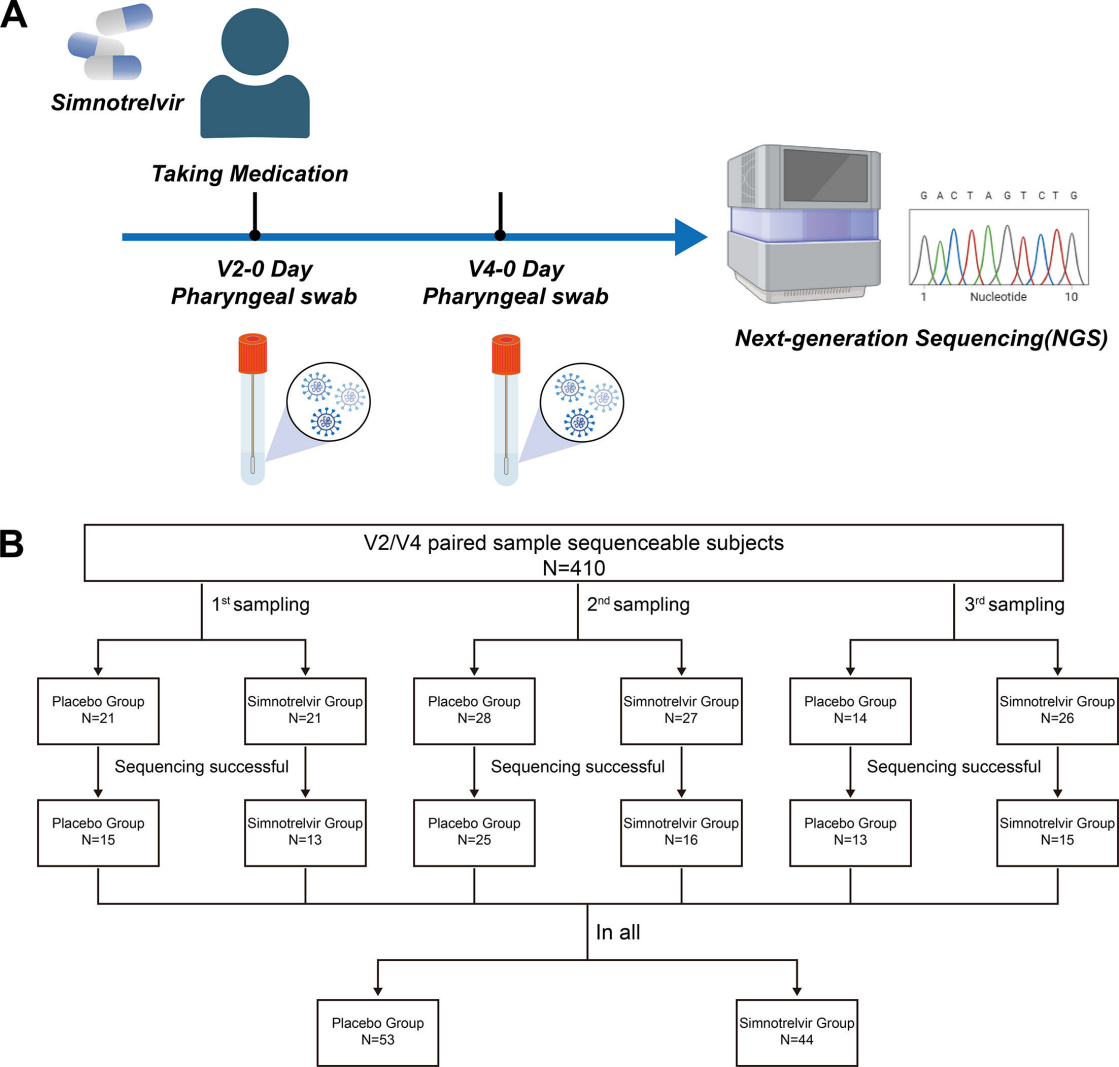
Clinical drug resistance experimental design and cohort summary. Viral nucleic acids were extracted from throat swab samples collected from participants in a previous clinical trial to obtain full virus genome sequences. (**A**) Study design for sample collection and viral nucleic acid sequencing in simnotrelvir clinical trial participants. (**B**) Workflow for screening and distribution of sequencing samples.

In the previous study, we have demonstrated that early administration of simnotrelvir plus ritonavir significantly shortened the time to symptom resolution in adult patients with COVID-19, with no evident safety concerns (10). To evaluate the potential for clinical resistance to simnotrelvir, we analyzed genome sequences of SARS-CoV-2 isolated from clinical samples from COVID-19 patients receiving simnotrelvir. Baseline and post-treatment viral sequencing data were available from 97 randomly selected patients, including 44 participants in the simnotrelvir plus ritonavir arm and 53 participants in the placebo arm (Fig. 4A and B). In the simnotrelvir-treated group, several mutated nucleotides within the 3CL^pro^ (nsp5 gene) were identified, including 393A > G, 395A > C, 561C > T, 688T > C, 771T > A, resulting in two amino acid mutations of P132H and F230L. In addition, the P132H mutation was found in one simnotrelvir-treated participant (2.3%) and in three placebo-treated participants (5.7%), while F230L mutation was observed in one simnotrelvir-treated participant (2.3%). Mutations in the 3CL^pro^ cleavage sites (nsp14/nsp15 and nsp15/nsp16 genes) included 1543G > T and 1000delA, corresponding to D515Y and C333fs amino acid mutations. These two mutations were each identified in one simnotrelvir-treated participant (2.3%) (Table 1). Notably, both F230L and D515Y mutations were detected in the same participant (Table S1), but neither contributed to resistance against 3CL^pro^ inhibitors. Furthermore, two participants were infected with the BA.5.2.48 and BA.5.2 variants, which contained a revert mutation and a missense mutation, respectively (Table S1). Interestingly, similar mutations were also identified in the placebo group. In summary, while simnotrelvir treatment might induce amino acid changes in the 3CL^pro^ region or its cleavage sites, no key mutations leading to 3CL^pro^ resistance were observed in our clinical cohort.

**TABLE 1:** Incidence of gene mutations after administration^*a*^

Gene mutation	Gene mutation site	Amino acid mutation site	3CL^pro^			3CL^pro^ cleavage site		
			S+R (*N* = 44) *n* (%)	Placebo (*N* = 53) *n* (%)	Total (*N* = 97) *n* (%)	S+R (*N* = 44) *n* (%)	Placebo (*N* = 53) *n* (%)	Total (*N* = 97) *n* (%)
nsp5	c.393A > G	p.R131R	1 (2.3%)	3 (5.7%)	4 (4.1%)	0	0	0
nsp5	c.395A > C	p.H132P	1 (2.3%)	3 (5.7%)	4 (4.1%)	0	0	0
nsp5	c.561C > T	p.D187D	0	1 (1.9%)	1 (1.0%)	0	0	0
nsp5	c.688T > C	p.F230L	1 (2.3%)	0	1 (1.0%)	0	0	0
nsp5	c.771T > A	p.T257T	1 (2.3%)	0	1 (1.0%)	0	0	0
nsp14/nsp15	c.1543G > T	p.D515Y	0	0	0	1 (2.3%)	0	1 (1.0%)
nsp15/nsp16	c.1000_1000delA	p.C333fs	0	0	0	1 (2.3%)	0	1 (1.0%)

^*a*^ S+R represents simnotrelvir + ritonavir.

## DISCUSSION

The outbreak of COVID-19, especially the constant variation of SARS-CoV-2, has brought great challenges to the development of vaccines and neutralizing antibodies (29–31). Recent studies have shown that antibodies in the serum of vaccine recipients and infected individuals exhibit significantly impaired neutralizing efficacy against newly emerging variants (15, 17, 31–33). In addition, multiple therapeutic monoclonal antibodies, targeting SARS-CoV-2 spike receptor-binding domain (RBD) (34–37) and non-RBD sites (38, 39), have largely lost their activity against these emerging variants (40). Nevertheless, oral small-molecule drugs have attracted increasing attention due to the wide range of activities and high stability. Currently, several anti-SARS-CoV-2 oral small-molecule drugs have been launched, including SARS-CoV-2 RNA polymerase inhibitor azvudine (41), molnupiravir (42), SARS-CoV-2 3CL^pro^ inhibitor nirmatrelvir (43), simnotrelvir (10), ensitrelvir (11), and leritrelvir (12). Notably, we previously reported that simnotrelvir has shown potent inhibitory activities against SARS-CoV-2 WIV04, Delta, and Omicron variants (10, 14). In response to the continuous evolution of Omicron, here we further validated the strong antiviral efficacy of simnotrelvir against emerging Omicron variants BA.1, BA.4, BA.5, CH.1.1, XBB.1.5, XBB.1.16, EG.5, and JN.1. In addition, 3CL^pro^ is highly conserved among known coronavirus species, and several common features are shared among the different coronavirus 3CL^pro^ substrates, making 3CL^pro^ an ideal target for specific antiviral therapies. Therefore, simnotrelvir, targeting 3CL^pro^, is a promising drug with both broad-spectrum and sustained therapeutic efficacy despite the persistent mutations of SARS-CoV-2 variants and the emergence of new coronaviruses.

The emergence of drug resistance has always been a critical issue in antiviral treatment (25). Although antiviral drugs targeting SARS-CoV-2 have been used for a short time, resistance has already been identified, mainly due to the mutations accumulating in the viral genome (44). Notably, SARS-CoV-2 3CL^pro^ resistance to nirmatrelvir has been shown to develop through multiple mechanisms, including reducing drug binding or increasing enzymatic activity (25). Nevertheless, our findings suggest that simnotrelvir may potentially ameliorate nirmatrelvir-resistant 3CL^pro^ mutations *in vitro*. Specifically, we showed that the 3CL^pro^ E166 mutation, which confers strong resistance to nirmatrelvir, remains relatively sensitive to simnotrelvir. And the crucial role of E166 in the interaction between SARS-CoV-2 3CL^pro^ and nirmatrelvir was elaborated in a recent study (45). Consistent with our results, another study also showed that the E166V mutation reduced the inhibitory potency of simnotrelvir, albeit to a lesser extent than that of nirmatrelvir (46). Although further studies are needed, these results suggested that simnotrelvir might have some advantages in treating SARS-CoV-2 3CL^pro^ E166V-resistant mutants. Furthermore, clinical data revealed that paxlovid treatment could lead to the emergence of SARS-CoV-2 E166 mutations strongly associated with drug resistance, emphasizing the potential of simnotrelvir in treating COVID-19 patients harboring the SARS-CoV-2 E166V mutation (16).

Examination of early BA.5 and coronavirus passages confirmed a gradual increase in simnotrelvir resistance with serial passages. Especially, our data showed that the inhibition potency of simnotrelvir to the investigated coronaviruses was not significantly compromised until 12 passages, indicating that simnotrelvir has a high barrier to develop drug resistance. Sequencing analysis of the clinical samples collected from treated patients showed that simnotrelvir did not induce the known resistant mutations in the 3CL^pro^ gene or its cleavage sites following drug administration, and the observed amino acid changes were not related to previously identified SARS-CoV-2 resistance mutations. Together, considering the *in vitro* selection test and clinical monitoring of viral resistant mutations, we anticipate that simnotrelvir will remain an effective antiviral agent against current circulating SARS-CoV-2 variants.

However, there are some limitations in our study. First, only two coronaviruses, Omicron BA.5 and HCoV-OC43, were used to assess the potential of simnotrelvir to elicit drug resistance. The recent emerging SARS-CoV-2 variants were not included in our analysis (27, 28). Second, the clinical monitoring of SARS-CoV-2 viral mutations was conducted on a relatively small sample size of simnotrelvir-treated patients.

In summary, simnotrelvir is an effective agent with high antiviral potency against SARS-CoV-2 Omicron and emerging variants. Our *in vitro* selection and clinical monitoring analysis suggested that the frequency of 3CL^pro^ associated resistance mutations is very low after treatment with simnotrelvir. These findings enhance our understanding of drug-induced resistance mechanisms and will lead the way for further optimization of current SARS-CoV-2 3CL^pro^ inhibitors.

## MATERIALS AND METHODS

### Cell lines and virus

Vero E6 cells and rhabdomyosarcoma cells (RD) were purchased from ATCC and cultured in Dulbecco’s modified Eagle medium (DMEM, Gibco) supplemented with 10% (vol/vol) fetal bovine serum (FBS, Gemini), 100 U/mL penicillin, and 100 mg/mL streptomycin.

SARS-CoV-2 Omicron variants, including BA.1 (EPI_ISL_7138045), BA.4 (EPI_ISL_13777657), BA.5 (EPI_ISL_13777658), XBB.1.5 (EPI_ISL_17205250), and CH.1.1 (EPI_ISL_17205252), were isolated from the University of Hong Kong. EG.5.1 (CG20230829-02), JN.1 (CG20231227-01), and XBB.1.16.1 (CG20230502-40) were isolated from Hubei Provincial Center for Disease Control and Prevention. HCoV-OC43 was isolated from the clinical samples. All experiments using authentic SARS-CoV-2 and its variants were carried out in a biosafety level 3 (BSL-3) facility.

### Compound and preparation

Simnotrelvir was provided by Jiangsu Simcere Pharmaceutical Co., Ltd. Nirmatrelvir and P-glycoprotein (P-gp) efflux inhibitor (CP-100356) were purchased from MCE. Then, the compound was dissolved in dimethyl sulfoxide (DMSO) to make a stock solution and aliquoted for storage at −80°C. Before the experiment, an aliquot of stock solution was diluted with DMEM to different concentration gradients.

### Antiviral activity assay

To evaluate the antiviral activity of simnotrelvir against the Omicron variants BA.1, BA.4, BA.5, CH.1.1, and XBB.1.5, vero E6 cells were seeded into 48-well plates at a density of 100,000 cells/well and incubated at 37°C in a 5% CO_2_ incubator. After 24 hours, the culture supernatants were removed. Subsequently, the serially diluted simnotrelvir and 0.5 µM P-gp inhibitor were added to the wells. The cells were then infected with SARS-CoV-2 Omicron variants at a multiplicity of infection (MOI) of 0.01 and incubated for 1 hour in a BSL-3 facility. Following the incubation, the supernatant was removed, and the cells were washed once with the medium before being treated with the fresh medium containing serially diluted simnotrelvir in the presence of the 0.5 µM P-gp inhibitor. The cells were then incubated for an additional 48 hours at 37°C in a 5% CO_2_ incubator. Then, the supernatants were collected, and viral RNA levels were quantified using real-time fluorescence quantitative PCR (RT-PCR). The concentration value (μM) and inhibition rate (%) of the test compound were entered into the X and Y columns in the GraphPad Prism V10.0 software, respectively. In the Analyze Data option, select the Transform concentration (X) program, select the transform to logarithms, and finally select the dose–response–inhibition slope (four parameters) program. The inhibition curves were obtained by four-parameter fitting, and then the absolute IC_50_ and IC_90_ of the compounds were obtained.

For the evaluation of the antiviral activity of the compounds against the Omicron variants XBB.1.16, EG.5, and JN.1, Vero E6 cells were seeded into 96-well plates at a density of 40,000 cells/well and incubated at 37°C in a 5% CO_2_ incubator. After 24 hours of incubation, the serially diluted simnotrelvir, in the presence of 0.5 µM P-gp inhibitor, was added to the wells. The cells were then infected with SARS-CoV-2 Omicron variants at a concentration of 100 TCID_50_/0.05 mL (TCID_50_: 50% tissue culture infectious dose) and incubated for 1 hour in a BSL-3 facility. The cells were washed once with the medium before being treated with the fresh medium containing the serially diluted simnotrelvir in the presence of CP-100356. The cells were then incubated for an additional 72 hours at 37°C in a 5% CO_2_ incubator. After incubation, Cell Titer-Glo reagents were added into cells according to the instruction. Cell viability was assessed using the Cell Titer-Glo chemiluminescence assay. The concentration value (μM) and inhibition rate (%) of the test compound were entered into the X and Y columns in the GraphPad Prism V10.0 software, respectively. In the Analyze Data option, select the Transform concentration (X) program, select the transform to logarithms, and finally select the dose–response–inhibition slope (three parameters) program. The inhibition curves were obtained by three-parameter fitting, and then the absolute IC_50_ and IC_90_ of the compound were obtained.

### RNA extraction and quantitative RT-PCR

Nucleic acid extraction of the 25 µL culture supernatant was performed by using the QIAamp Viral RNA Mini Kit (Qiagen, 52906) according to the manufacturer’s instruction. The final elution volume of RNA was 60 µL. As previously described (47), the primers and probe (IDT, 104282139) used were designed to detect the SARS-CoV-2 RdRp-Hel gene. The Quantinova Probe RT-PCR kit (Qiagen, 208356) was used in the StepOne Real-time PCR system. The thermal cycling condition was 10 minutes at 45°C for reverse transcription, 5 minutes at 95°C for PCR initial activation, and 45 cycles of 5 seconds at 95°C and 30 seconds at 55°C.

### Cell viability assays

Cell viability in drug-treated wells was assessed using the Cell Titer-Glo Luminescent Cell Viability assay kit (Promega, G7570) as the manufacturer’s protocol. The assay signal (luminescence) was collected using a LumiStation 1800 Chemiluminescence Microplate Reader (Shanghai Flashpu Biotechnology Co., Ltd).

### Enyzmatic inhibition of simnotrelvir against nirmatrelvir-resistant 3CL^pro^ mutants

Wild-type 3CL^pro^ and the six SARS-CoV-2 3CL^pro^ mutants with varying degrees of resistance to nirmatrelvir, including A260V, Y54A, (T21I + S144A), F140A, H172Y, and E166V, were cloned and expressed by WuXi AppTec Shanghai Drug Development Co., Ltd. To evaluate the inhibitory activity of simnotrelvir against nirmatrelvir-resistant 3CL^pro^ mutants, the 3CL^pro^ enzymatic inhibition assay was performed by fluorescence resonance energy transfer (FRET). The fluorogenic substrate DABCYL-KTSAVLQSGFRKME-Edans (GenScript, Cat: C005PE290-5/PE4945) can be cleaved by 3CL^pro^ and generates an Edans peptide fragment that emits intense fluorescence signal at 340 nm (excitation) /490 nm (emission).

The FRET-based enzymatic assay was performed as follows. First, 25 µL of each 3CL^pro^ mutant was incubated with 25 µL of assay buffer (20 mM Tris-HCl pH 7.3, 100 mM NaCl, 1 mM EDTA, 5 mM TCEP, 0.1% BSA) containing the serially diluted simnotrelvir or nirmatrelvir for 30 minutes. The reaction was initiated by adding 5 µL of the fluorogenic substrate in 384-well plates. To determine the IC_50_ values, threefold serially diluted drugs were used in each experiment with triplicate wells. More details of the experiment are provided in Table S2. The inhibition curves were obtained by four-parameter fitting, as described before, and then the relative IC_50_ values of the compound were obtained.

### *In vitro* selection for BA.5 variant resistance in Vero E6 cells

The BA.5 variant is serially passaged under the selective pressure of simnotrelvir to induce potential resistance mutations, with IC_50_ values measured across different viral passages to determine the development of a resistant phenotype.

In detail, Vero E6 cells are seeded at 200,000 cells/mL in 12-well plates and incubated at 37°C in CO₂ for 16 hours. After a 1 hour incubation with the diluted compounds, cells are infected with the BA.5 variant (P0) at an MOI of 0.01. Subsequent viral challenges used 20 µL of the viral supernatant from the previous generation. Plates are sealed and incubated for 72 hours at 37°C.

The assay was performed by monitoring the BA.5 variant on infected Vero E6 cells to determine the IC_50_ values of simnotrelvir and changes in drug susceptibility. Vero E6 cells were seeded in 48-well plates at a density of 50,000 cells/well and incubated at 37°C in a CO₂ incubator until 90% confluence was reached. The supernatant was then removed, and 200 µL of diluted simnotrelvir or nirmatrelvir in 2% FBS-DMEM was added to each well. After a 1 hour incubation at 37°C, the cells were infected with BA.5 at an MOI of 0.01 using the corresponding viral generation. Following a 72 hour incubation, cell viability in drug-treated wells is assessed using the Cell Titer-Glo Luminescent Cell Viability assay kit (Promega, G7570) as the manufacturer’s protocol. The inhibition curves were obtained by four-parameter fitting as described before, and then the absolute IC_50_ values of the compound were obtained.

### *In vitro* selection for HCoV-OC43 resistance in RD cells

HCoV-OC43 was serially passaged under the selective pressure of simnotrelvir to induce potential resistance mutations, with IC_50_ values measured across different viral passages to determine the development of a resistant phenotype.

Rhabdomyosarcoma (RD) cells were seeded at 200,000 cells/mL in 12-well plates and incubated at 37°C in CO₂ for 16 hours. After a 1 hour incubation with the diluted compounds, cells were infected with the HCoV-OC43 wild-type strain (P0) at an MOI of 0.1. Subsequent viral challenges used 20 µL of the viral supernatant from the previous generation. Plates were sealed and incubated for 72 hours at 37°C.

The assay was performed by monitoring HCoV-OC43 on infected RD cells to determine the IC_50_ values of simnotrelvir and changes in drug susceptibility. RD cells were seeded in 48-well plates at a density of 50,000 cells/well and incubated at 37°C in a CO₂ incubator until 90% confluence was reached. The supernatant was then removed, and 200 µL of diluted simnotrelvir or nirmatrelvir in 2% FBS-DMEM was added to each well. After a 1 hour incubation at 37°C, the cells were infected with HCoV-OC43 at an MOI of 0.1 using the corresponding viral generation. Following a 48 hour incubation, RNA was extracted from the cells, and qPCR was performed to quantify viral RNA. The IC_50_ values of simnotrelvir or nirmatrelvir were calculated for the 12 passages of virus infection. The inhibition curves were obtained by four-parameter fitting, as described before, and then the relative IC_50_ values of the compound were obtained.

### Clinical drug resistance study of simnotrelvir

In a multicenter, randomized, double-blind, phase II/III clinical study aiming at evaluating the efficacy and safety of simnotrelvir oral administration in symptomatic mild-to-moderate COVID-19 adult subjects (10), a total of 1,200 volunteers participated and were randomly assigned to two groups, with 600 participants in each group. One group received oral simnotrelvir plus ritonavir, while the other group received a placebo (adjuvant only).

This study sampled approximately 200 previously collected nucleic acid samples from 100 subjects (50 from the treatment group and 50 from the placebo group). Samples were obtained from throat swabs taken during V2 (Day 0) and V4 (Day 5 post-treatment). To explore the relationship between viral mutations and clinical outcomes, sample selection was based on the following criteria: (1) viral load greater than 2.86E + 03 at V2 and V4, making the clinical samples feasible for sequencing. (2) Presence of viral variants. (3) Recurrent positive patients who tested positive for SARS-CoV-2 antigen or nucleic acids.

Viral gene sequencing was performed on these prioritized samples. Throat swabs were processed for viral RNA extraction, and full gene sequences were obtained using next-generation sequencing (NGS). Sequencing results from Visit 2 and Visit 4 were compared to identify amino acid changes and mutations in the 3CL^pro^ gene and its cleavage sites.

## SUPPLEMENTAL MATERIAL

Supplemental tables aac.01556-24-s0001.docx Tables S1 and S2.

ASM does not own the copyrights to Supplemental Material that may be linked to, or accessed through, an article. The authors have granted ASM a non-exclusive, world-wide license to publish the Supplemental Material files. Please contact the corresponding author directly for reuse.
