# Supplementary material for: Potent antiviral activity of simnotrelvir against key epidemic SARS-CoV-2 variants with a high resistance barrier
Source: Antimicrob Agents Chemother. 2025 Mar 10;69(4):e01556-24. doi: 10.1128/aac.01556-24 (PMC11963564; doi:10.1128/aac.01556-24)
Supplement: Supplemental tables — Tables S1 and S2. [file aac.01556-24-s0001.docx]

**Supplementary Table 1 The list of gene mutations after administration**

| Gene mutation type | No. | Group | Variant  Type | Gene mutation site (target gene starting calculation) | Abbreviation of amino acid mutation site (target protein starting calculation) | Nucleotide mutation type | Reverse mutation | Recurrent positive |
| --- | --- | --- | --- | --- | --- | --- | --- | --- |
| 3CL^pro^ | 301023285 | (S+R) | BA.5.2.48 | c.395A>C | p.P132H | Missense mutations | YES | NO |
|  |  |  |  | c.393A>G | p.R131R | Synonymous mutations | YES |  |
| 3CL^pro^ | 301067003 | (S+R) | BA.5.2.49 | c.688T>C | p.F230L | Missense mutations | NO | YES |
| 3CL^pro^ | 301023117 | (S+R) | BA.5.2 | c.771T>A | p.T257T | Synonymous mutations | NO | YES |
|  |  |  |  |  |  |  |  |  |
| 3CL^pro^ | 301009267 | Placebo | BA.5.2.48 | c.395A>C | p.P132H | Missense mutations | YES | NO |
|  |  |  |  | c.393A>G | p.R131R | Synonymous mutations | YES |  |
| 3CL^pro^ | 301023347 | Placebo | BF.7.14 | c.395A>C | p.H132P | Missense mutations | YES | NO |
|  |  |  |  | c.393A>G | p.R131R | Synonymous mutations | YES |  |
| 3CL^pro^ | 301064020 | Placebo | BA.5.2.1 | c.395A>C | p.P132H | Missense mutations | YES | YES |
|  |  |  |  | c.393A>G | p.R131R | Synonymous mutations | YES |  |
| 3CL^pro^ | 301009185 | Placebo | BA.5.2 | c.561C>T | p.D187D | Synonymous mutations | NO | NO |
| 3CL^pro^ cleavage site | 301023116 | (S+R) | BA.5.2 | c.1000_1000delA | p.C333fs | Frame shift mutations | NO | NO |
| 3CL^pro^ cleavage site | 301067003 | (S+R) | BA.5.2.49 | c.1543G>T | p.D515Y | Missense mutations | NO | YES |

S+R represents Simnotrelvir +Ritonavir

**Supplementary Table 2 3CL^pro^ protease and corresponding substrate test concentration information and reaction time**

| WT or mutant  3CL^pro^ proteins | Final concentration  of protease (nM) | Final concentration  of substrate (μM) | Reaction time (min) |
| --- | --- | --- | --- |
| Wildtype | 50 | 20 | 60 |
| A260V | 25 | 12.5 | 60 |
| Y54A | 100 | 25 | 120 |
| T21I+S144A | 25 | 25 | 60 |
| F140A | 25 | 12.5 | 60 |
| H172Y | 100 | 25 | 120 |
| E166V | 100 | 25 | 120 |
